# Supplementary material for: Bradyzoite subtypes rule the crossroads of Toxoplasma development
Source: Nat Commun. 2026 Jan 24;17:1783. doi: 10.1038/s41467-026-68489-y (PMC12917143; doi:10.1038/s41467-026-68489-y)
Supplement: Supplementary file 1 — Supplementary Information [file 41467_2026_68489_MOESM1_ESM.pdf]

Supplementary Figure 1

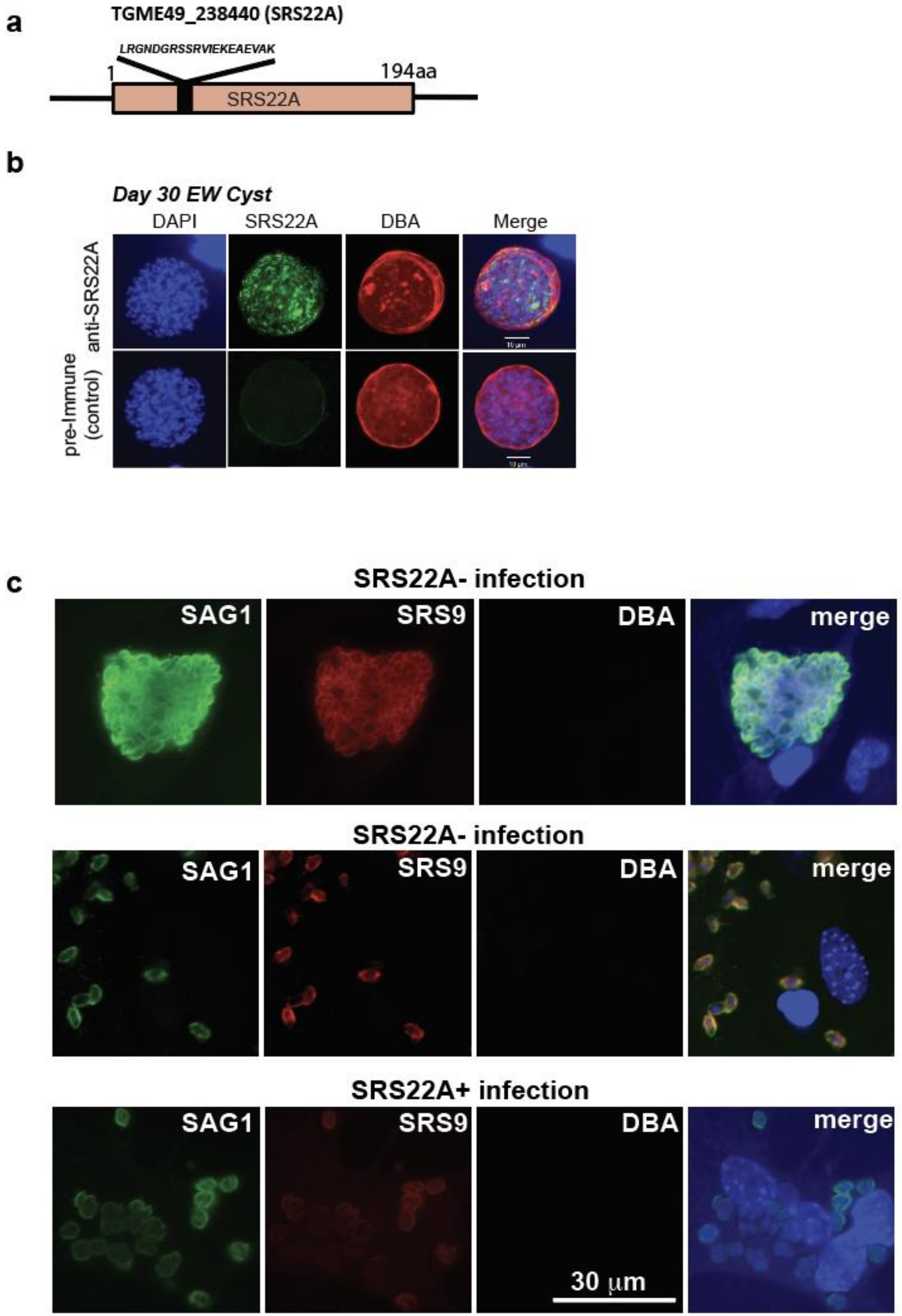

Supplementary Figure 1. Generation and validation of an SRS22A antibody, and its application in astrocytes. a Peptide selected for generating a monoclonal SRS22A antibody in rabbit. **b** Immunofluorescence images of co-staining of ME49EW cyst using rabbit-a-SRS22A antibody (green), pre-immune control (green) and DBA (red). **c** Examples of intermediate staining of parasite vacuoles (SAG1-green and SRS9-red) in FACS sorted SRS22A negative infections in astrocytes 5-days post-infection after FACS sorting (top panel), and examples of secondary infections in both FACS sorted SRS22A + and – infections in astrocytes on 5-days post-infection following FACS sorting.

Supplementary Figure 2

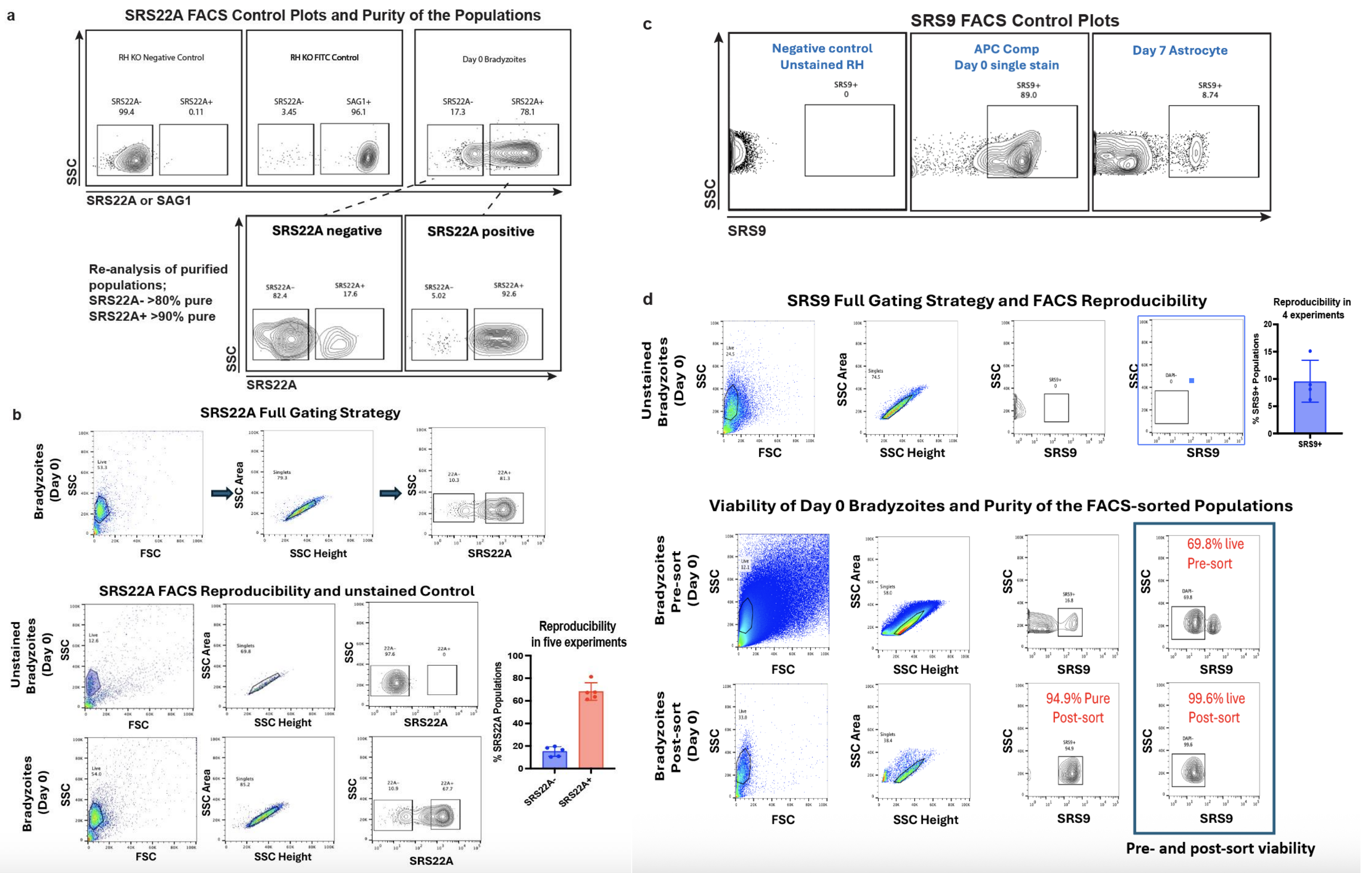

Supplementary Figure 2. FACS sorting of bradyzoites using SRS22A antibody. a and b Compensation controls, full gating strategy used in FACS purification as well as the reproducibility and purity of SRS22A positive and negative populations from 40-day infected mouse brains (**Panels a and b**) and SRS9 positive and negative populations (**Panels c and d**) from *ex vivo* Day 7 cultures from astrocytes infected with *ex vivo* bradyzoites. Source data are provided as a Source Data file for this figure.

Supplementary Figure 3

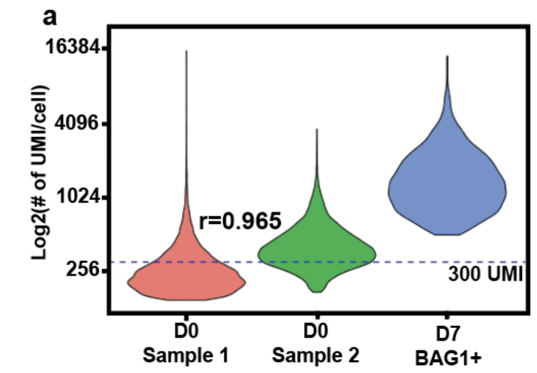

b

Library	D0-1	D0-2	Day-7 Astro
Total reads (x10^8)	7.24	15.4	8.76
Mapped reads (fraction reads in cells)	81.40%	82.50%	80.60%
# Cells	8,609	9,153	29,931
Mean reads/cell	84,205	168,724	32,523
Median reads/cell	177	267	511
Total genes detected	7,705	7,978	8,115
> 300 UMI cell counts	888	3,302	26,414

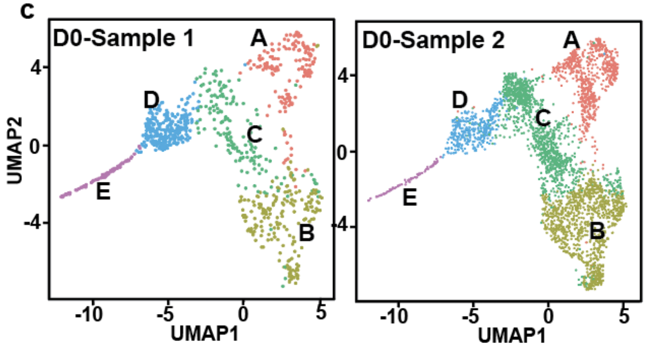

d

Selected growth gene comparison.
GRA1 and GRA2 are consitutive controls

Gene ID TgME49_	Gene name	Grp A **20%	Grp B 30%	Grp C 31%	Grp D 15%	Grp E 4%
270250	GRA1	3.4	4.8	3.7	2.5	1.4
227620	GRA2	2.8	3.9	3.1	2.2	1.7
209910	H2B	2.2	1.1	1.5	1.8	3.6
300200	H2A	1.5	1.2	1.3	1.4	3.0
316400	aTUB	1.4	0.4	0.7	1.5	3.8
266960	bTUB	1.2	0.6	1.0	2.3	3.4
250340	Centrin 2	0.2	0.07	0.01	0.2	2.0
260820	ISP1	1.1	0.3	0.3	0.4	2.8
231640	IMC1	1.5	0.6	0.6	0.3	2.1
216000	IMC3	1.2	0.2	0.2	0.09	2.4

**% of total bradyzoites sequenced and >300 UMI (4,181 total Bz).

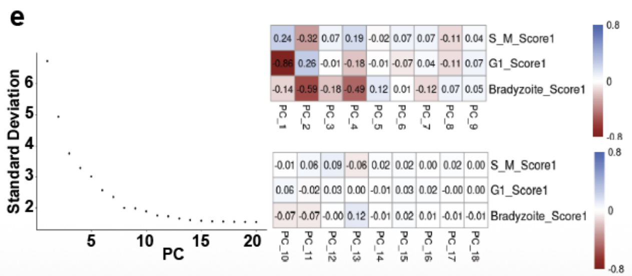

f

Comparison of early in vitro bradyzoites (D7-BAG1+) to in vivo bradyzoites (A-E)

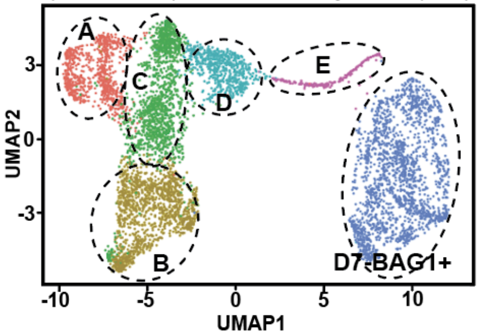

Gene numbers per bradyzoite Group A-E compared to early D7-BAG1+ bradyzoites

Group	#up-reg	#down-reg	#unique up-reg
A	401	243	109
B	385	543	103
C	298	264	67
D	359	278	32
E	495	259	173

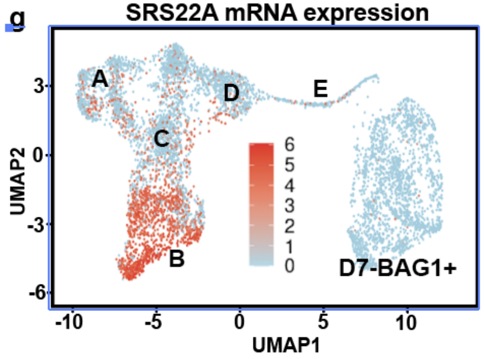

Supplementary Figure 3. Quality control, reproducibility, and cell cycle/ growth genes-related analyses of scRNA-seq data. a Number of unique molecular identifiers (UMIs) per cell is shown for each sample included in the analysis. A 300 UMI threshold cutoff was applied to all samples to filter out poor quality parasites. We also performed a correlation analysis between the two *in vivo* bradyzoite samples (excysted bradyzoites from 40-day infected mice tissue cysts) included in the scRNAseq analysis. A strong correlation was observed, Pearson correlation coefficient of r=0.965. **b** Quality assurance parameters of scRNA seq are shown. **c** UMAP projections of bradyzoites from two independent harvests of 40-day brain tissue cysts show identical clustering. **d** Table of selected growth gene expression (average expressed values) across Group A-E bradyzoites. Note that growth gene expression was uniformly higher in Group E bradyzoites, which constitutes 4% of the total bradyzoites analyzed in this experiment (4,181 total bradyzoites). The proportional value of the other bradyzoite groups is also indicated. **e** The elbow plot indicates a noticeable decrease in variation after principal component 18. Pearson correlations between cell embeddings from PCs 1-18 PCs and cell scores for G1, S/M and bradyzoite specific gene sets are shown. **f** UMAP projection of Groups A-E as compared to the BAG1+ bradyzoites from *ex vivo* bradyzoite-infected astrocytes at day 7. A distinct clustering of Groups A-E from the D7-BAG1+ parasites is easily seen. The Table below shows the total number of down and upregulated genes as well as the unique upregulated genes in each Group A-E. **g** UMAP projection displaying SRS22A expression shading shows a SRS22A expression is enhanced in Group B bradyzoites, while absent from D7-BAG1+ bradyzoites.

Supplementary Figure 4

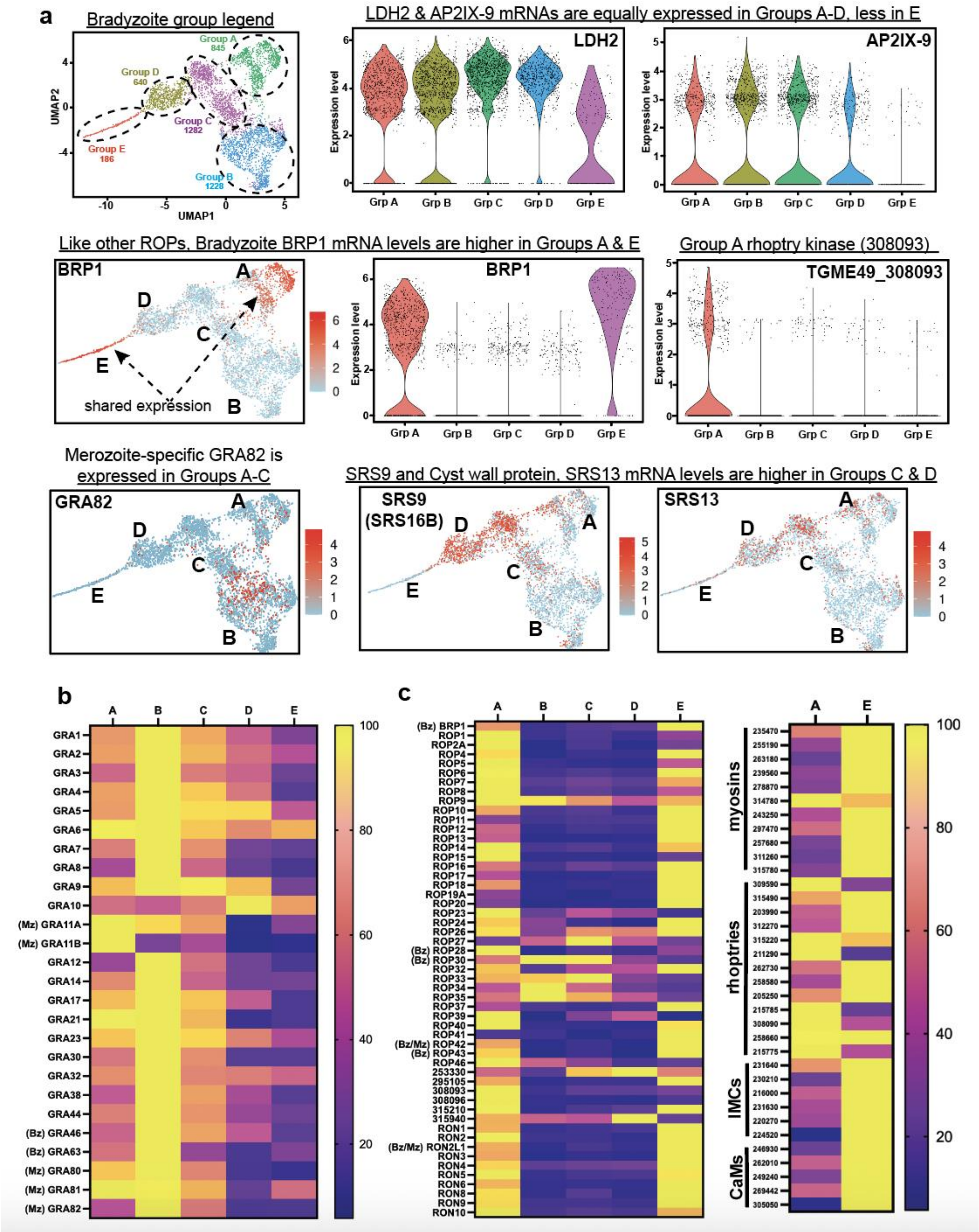

Supplementary Figure 4. UMAP projections and heatmaps of Groups A-E cluster specific genes in scRNA-seq data. a (top row) UMAP projection of *in vivo* bradyzoites references Groups A-E distributions. Violin plots of LDH2 and AP2IX-9 are shown for Groups A-E. (**second row**) UMAP mRNA shading of bradyzoite-specific rhoptry protein, BRP1, and associated violin plot are shown. The last panel shows an example of unique rhoptry gene expression (TGME49_308093) in Group A bradyzoites. (**third row**) UMAP projection of GRA82 mRNA levels showing enhanced expression in Groups A-C (3-7-fold higher than Groups D & E, Database S1), and UMAP images of SRS9 mRNA and cyst wall protein, SRS13 mRNA enhanced expression in Groups C and D bradyzoites. A heatmap of GRA **(b)** and ROP/RON **(c)** family gene expression across Groups A-E bradyzoites. The right heat map in (C) shows the analysis of representative S/M transcripts in Group A versus E bradyzoites. Note the increase of rhoptry mRNA expression in Group E is accompanied by increased expression of other S/M transcripts, while enhanced rhoptry mRNA expression of Group A bradyzoites did not show this cell cycle pattern.

Supplementary Figure 5

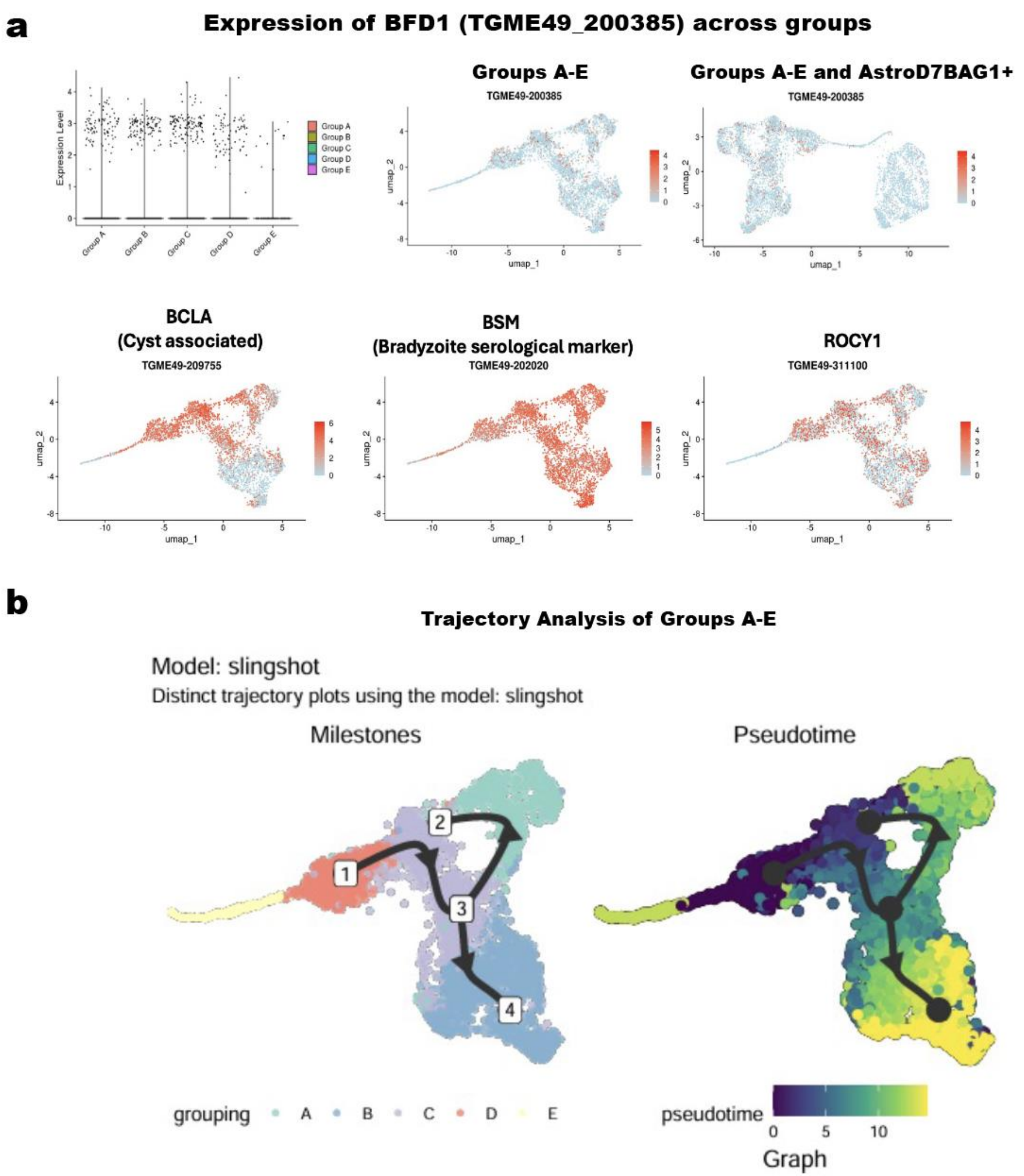

Supplementary Figure 5. Visualization of BFD1-related genes and trajectory analysis of all bradyzoite clusters. a Distribution of transcription factor BFD1 and BFD1-associated genes BCLA, BSM and ROCY1 across all bradyzoite clusters. **b** Developmental trajectory of the clusters was shown on the existing UMAPs. Branching after Group C/D transition parasites indicates a decision point towards either Group A or Group B.
