# Supplementary material for: Bradyzoite subtypes rule the crossroads of Toxoplasma development
Source: Nat Commun. 2026 Jan 24;17:1783. doi: 10.1038/s41467-026-68489-y (PMC12917143; doi:10.1038/s41467-026-68489-y)
Supplement: Supplementary file 2 — Description Of Additional Supplementary File [file 41467_2026_68489_MOESM2_ESM.pdf]

Description of Additional Supplementary Files

Title: Supplementary data 1
Description: scRNAseq processed data gene list.

Title: Supplementary data 2
Description: scRNAseq processed data gene list, separated by Groups A-E.
